# Supplementary material for: Motor neurons are dispensable for the assembly of a sensorimotor circuit for gaze stabilization
Source: bioRxiv. 2024 Jan 27:2024.01.25.577261. Preprint. [Version 1] doi: 10.1101/2024.01.25.577261 (PMC10849732; doi:10.1101/2024.01.25.577261)
Supplement: 1 [file NIHPP2024.01.25.577261V1-supplement-1.pdf]

bioRxiv preprint doi: https://doi.org/10.1101/2024.01.25.577261; this version posted January 27, 2024. The copyright holder for this preprint (which was not certified by peer review) is the author/funder, who has granted bioRxiv a license to display the preprint in perpetuity. It is made available under aCC-BY 4.0 International license.

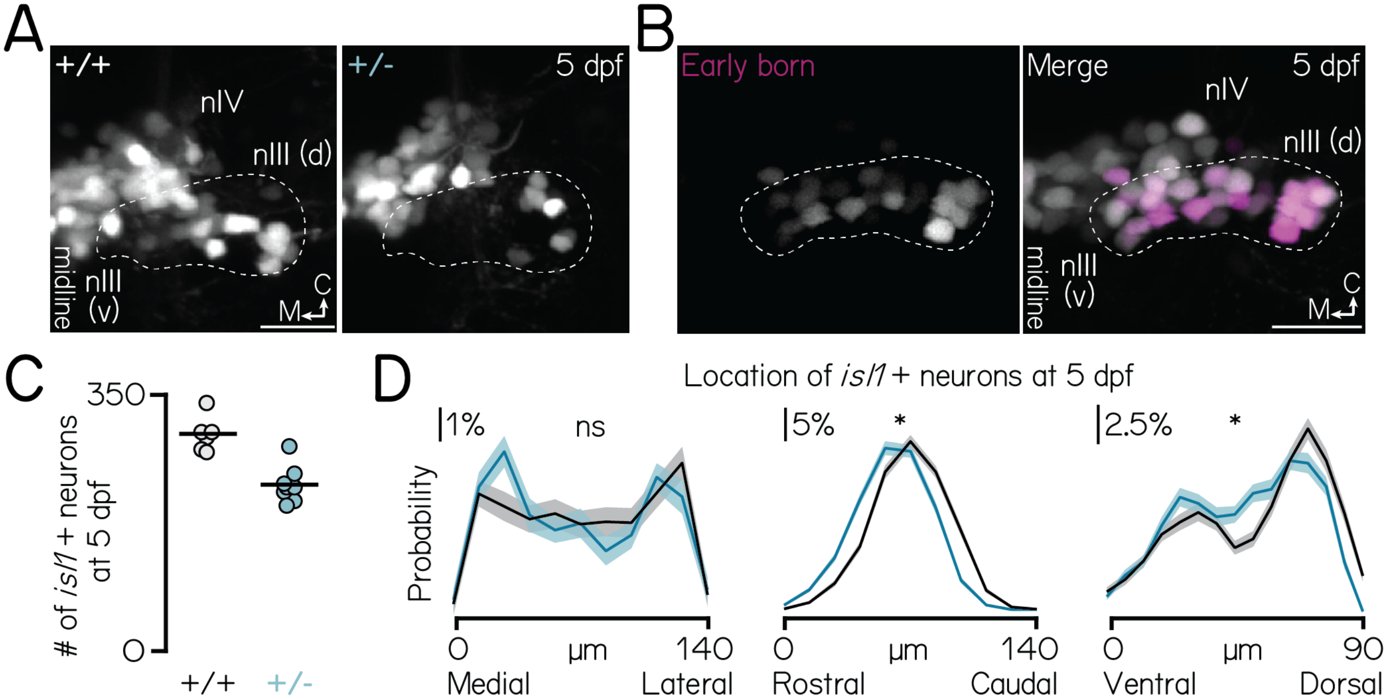

Figure S1: *phox2a* specifies nIII motor neuron fate in a dose- and birthdate-dependent manner.
Associated with Figure 1.
(A) Images of nIII/nIV motor neurons, labeled in *Tg(isl1:GFP)*, in wildtype siblings (left) and *phox2a* heterozygotes (middle) at 5 dpf. Wildtype image same as in Figure 1E. One hemisphere shown. White dashed lines outline the dorsal extent of nIII, which contains inferior rectus and medial rectus neurons[33]. Scale bar, 20 μm.
(B) Location of the earliest-born neurons in nIII/nIV (left, magenta) against all nIII/nIV neurons labeled in *Tg(isl1:Kaede)* (right, grey). Larvae birthdated at 34 hpf (Methods). One hemisphere shown. White dashed lines outline the dorsal extent of nIII. Scale bar, 20 μm.
(C) Quantification of the number of *Tg(isl1:GFP)*+ neurons in nIII/nIV from N=6 wildtype siblings (grey) and N=8 *phox2a* heterozygotes (teal). Wildtype data same as Figure 1F.
(D) Distributions showing probability of nIII/nIV soma location across each spatial axis in wildtype (black) and heterozygous (teal) *phox2a* larvae. Solid and shaded lines show mean and standard deviation, respectively, from bootstrapped data. Data from same fish quantified in Figure S1C. ns, not significant; star, significant at the p<0.001 level.

bioRxiv preprint doi: https://doi.org/10.1101/2024.01.25.577261; this version posted January 27, 2024. The copyright holder for this preprint (which was not certified by peer review) is the author/funder, who has granted bioRxiv a license to display the preprint in perpetuity. It is made available under aCC-BY 4.0 International license.

A

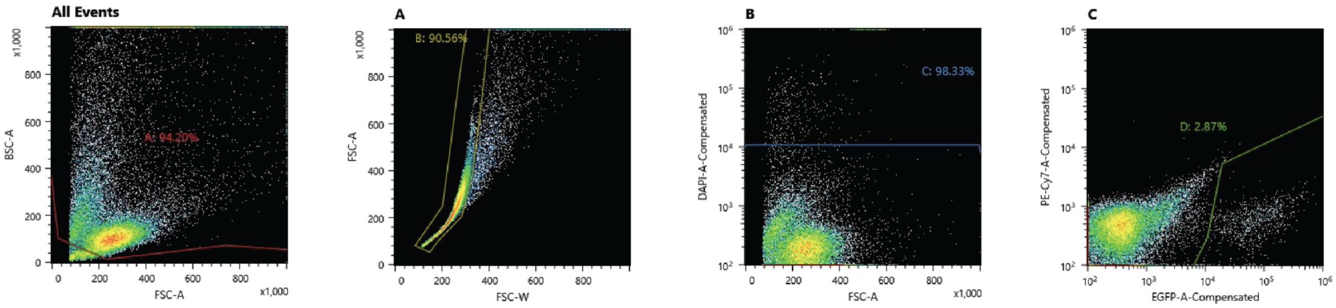

Figure S2: Flow cytometry gating strategy to sort fluorescently-labeled neurons for bulk RNA sequencing.
Associated with Figure 5.
(A) Sequential gates used to sort fluorescent neurons labeled with *Tg(-6.7Tru.Hcrtr2:GAL4-VP16)*;*Tg(UAS-E1b:Kaede)*;*Tg(isl1:GFP)*. Gate A excluded presumptive debris (small cells). Gate B isolated single cells and excluded large cells and doublets. Gate C excluded DAPI+ (dead or unhealthy) neurons. Gate D isolated fluorescent (GFP or Kaede+) neurons; neurons in this gate were sorted. Gates were set using negative controls (not shown; Methods). Gates shown for one of four experimental repeats.

bioRxiv preprint doi: https://doi.org/10.1101/2024.01.25.577261; this version posted January 27, 2024. The copyright holder for this preprint (which was not certified by peer review) is the author/funder, who has granted bioRxiv a license to display the preprint in perpetuity. It is made available under aCC-BY 4.0 International license.

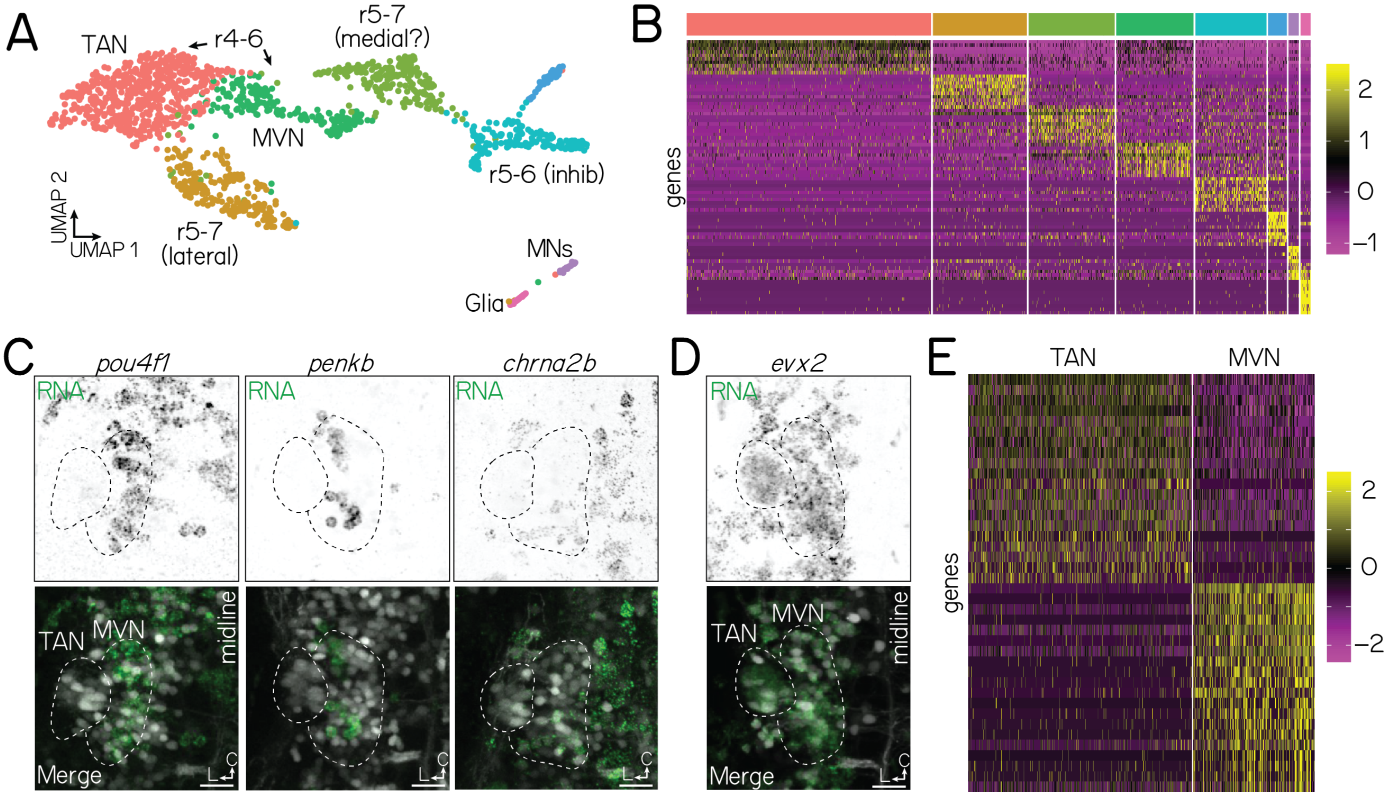

Figure S3: Molecular identification of projection neurons using a reference single-cell RNA sequencing atlas.
Associated with Figure 5.
(A) UMAP visualization of a single-cell RNA sequencing atlas of n=1,468 neurons labeled in *Tg(-6.7Tru.Hcrtr2:GAL4-VP16);Tg(UAS-E1b:Kaede)*, generated with 10x Genomics (Methods). Each circle is a single neuron. Neurons are clustered (colors) according to their transcriptional identity. Annotations are based on validated marker genes (data not shown). TAN, tangential nucleus; MVN, medial vestibular nucleus; r, rhombomere; MNs, motor neurons; inhib, inhibitory neurons.
(B) Heatmap showing genes unique to each annotated cluster. Each row is a gene; names unlisted for clarity. Columns show distinct clusters. Color bar on top reflects clusters in Figure S3A. Yellow and purple reflect stronger or weaker gene expression, respectively.
(C) Fluorescent *in situ* hybridization against three markers (*pou4f1, penkb, chrna2b*) that are negative for tangential nucleus projection neurons and positive for medial vestibular nucleus neurons. Top row shows RNA expression (green); bottom row, merge with neurons labeled in *Tg(-6.7Tru.Hcrtr2:GAL4-VP16);Tg(UAS-E1b:Kaede)*. Dashed lines outline the tangential nucleus (TAN) and medial vestibular nucleus (MVN). Data from 72 hpf larvae. Images shown in an axial view.
(D) Fluorescent *in situ* hybridization against a positive marker (*evx2*) for both tangential nucleus and medial vestibular nucleus neurons. All scale bars, 20 μm.
(E) Heatmap showing genes unique to tangential and medial vestibular neurons. Clusters identified using positive and negative fluorescent *in situ* data from Figure S3C-Figure S3D and unpublished data.

bioRxiv preprint doi: https://doi.org/10.1101/2024.01.25.577261; this version posted January 27, 2024. The copyright holder for this preprint (which was not certified by peer review) is the author/funder, who has granted bioRxiv a license to display the preprint in perpetuity. It is made available under aCC-BY 4.0 International license.

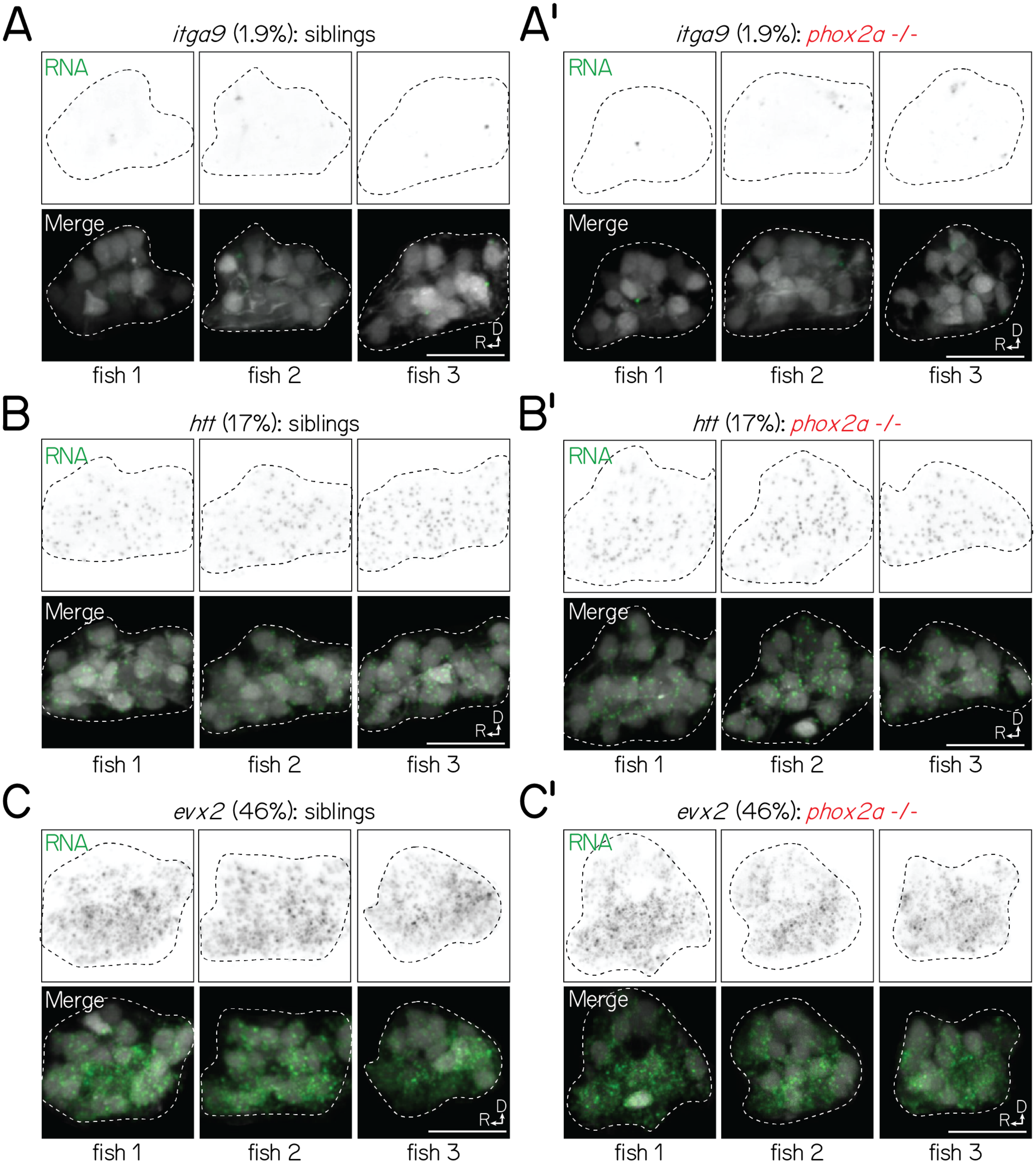

Figure S4: Visualization of transcripts in siblings and *phox2a* null mutants with fluorescent *in situ* hybridization is (1) consistent across larvae and (2) scales with predicted detection in projection neurons.
Associated with Figure 5.
(A-A') Fluorescent *in situ* hybridization against *itga9* for three sibling (A) or *phox2a* null mutant (A') larvae (72 hpf), imaged with identical conditions. Left column shows RNA (green); right column, merge with projection neurons visualized with *Tg(-6.7Tru.Hcrtr2:GAL4-VP16);Tg(UAS-E1b:Kaede)* (grey). Dashed lines outline the projection nucleus. Cell and transcript expression outside the projection nucleus is removed for visual clarity. Percentage (1.9%) refers to fraction of cells in a single-cell RNA sequencing reference atlas (Methods) with detected transcript. All scale bars, 20 μm.
(B-B') Fluorescent *in situ* hybridization against *htt*, 17%, for three sibling (B) and *phox2a* mutant (B') larvae (72 hpf).
(C-C') Fluorescent *in situ* hybridization against *evx2*, 46%, for three sibling (C) and *phox2a* mutant (C') larvae (72 hpf).

bioRxiv preprint doi: https://doi.org/10.1101/2024.01.25.577261; this version posted January 27, 2024. The copyright holder for this preprint (which was not certified by peer review) is the author/funder, who has granted bioRxiv a license to display the preprint in perpetuity. It is made available under aCC-BY 4.0 International license.

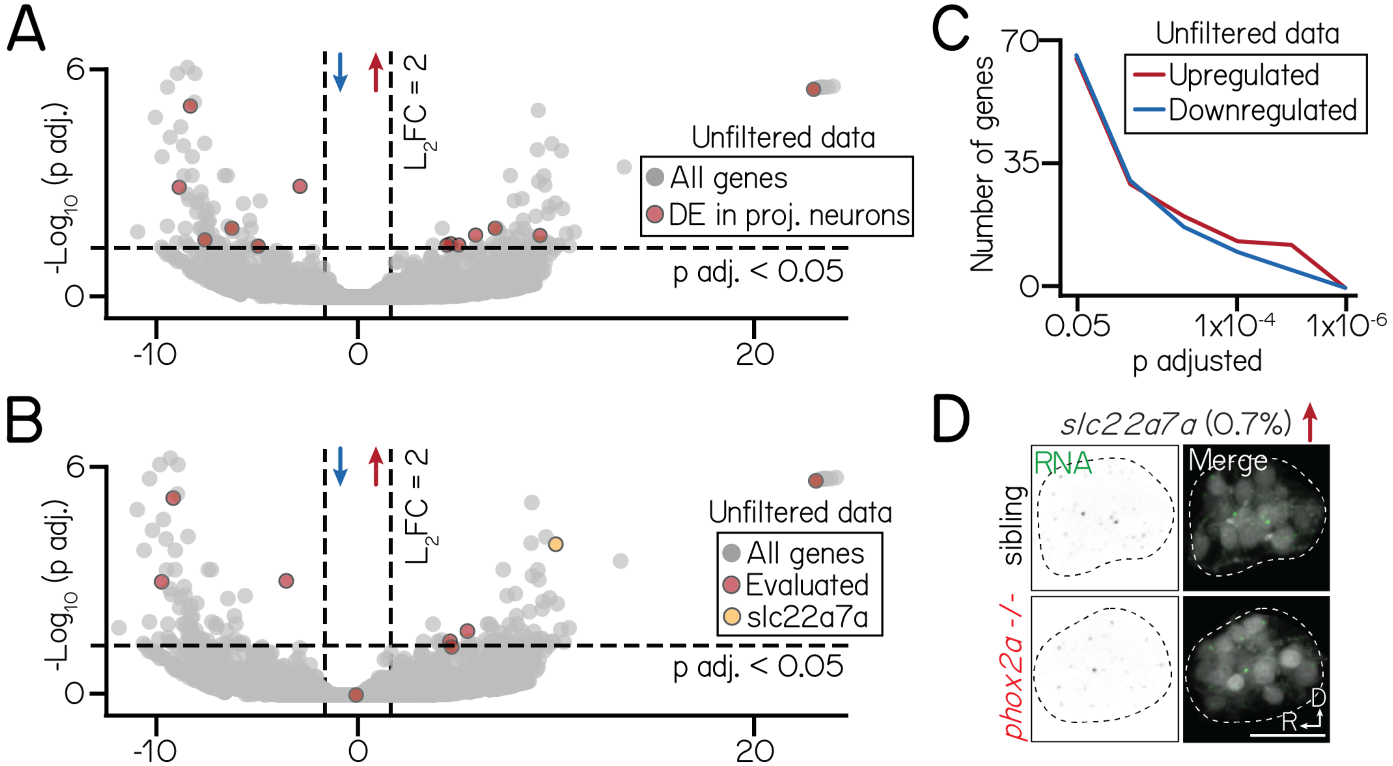

Figure S5: Differential gene expression in an unfiltered bulk sequencing dataset of siblings and *phox2a* mutants.
Associated with Figure 5.
(A) Volcano plot showing differentially expressed genes across an unfiltered bulk RNA sequencing dataset. Dashed lines represent significance cutoffs: horizontal line, p adjusted>0.05; vertical line, Log_2 Fold Change > 2.0. Each circle is a gene. Genes to the left and right of 0 on the horizontal axis show downregulated and upregulated genes, respectively. Red color shows genes that are differentially expressed in a filtered subset of projection neurons (Figure 5). Grey-colored genes are below both significance thresholds.
(B) Same data as Figure S5A, now highlighting candidate genes evaluated by fluorescent *in situ* (Figure 5) with red. One candidate (yellow) that did not meet projection neuron filter criteria (Methods) is shown in Figure S5D; remaining candidates (included in filtered data) shown in Figure 5F.
(C) Same data as Figure S5A-Figure S5B, showing the number of differentially expressed genes at progressive significance thresholds (p adjusted). Red and blue lines show the number of significantly upregulated and downregulated genes, respectively.
(D) Fluorescent *in situ* hybridization against a candidate gene, *slc22a7a* (log_2 fold change=10.2, p adj.=1.6×10^{-4}), that did not meet projection neuron filter criteria. Percentage refers to fraction of projection neurons from a single-cell sequencing dataset with expression (Methods). Left columns show RNA (green); right columns, merge with projection neurons labeled with *Tg(-6.7Tru.Hcrtr2:GAL4-VP16);Tg(UAS-E1b:Kaede)* (grey). Dashed lines outline the projection nucleus. Cell and transcript expression outside the projection nucleus is removed for visual clarity. All scale bars, 20 μm.

bioRxiv preprint doi: https://doi.org/10.1101/2024.01.25.577261; this version posted January 27, 2024. The copyright holder for this preprint (which was not certified by peer review) is the author/funder, who has granted bioRxiv a license to display the preprint in perpetuity. It is made available under aCC-BY 4.0 International license.

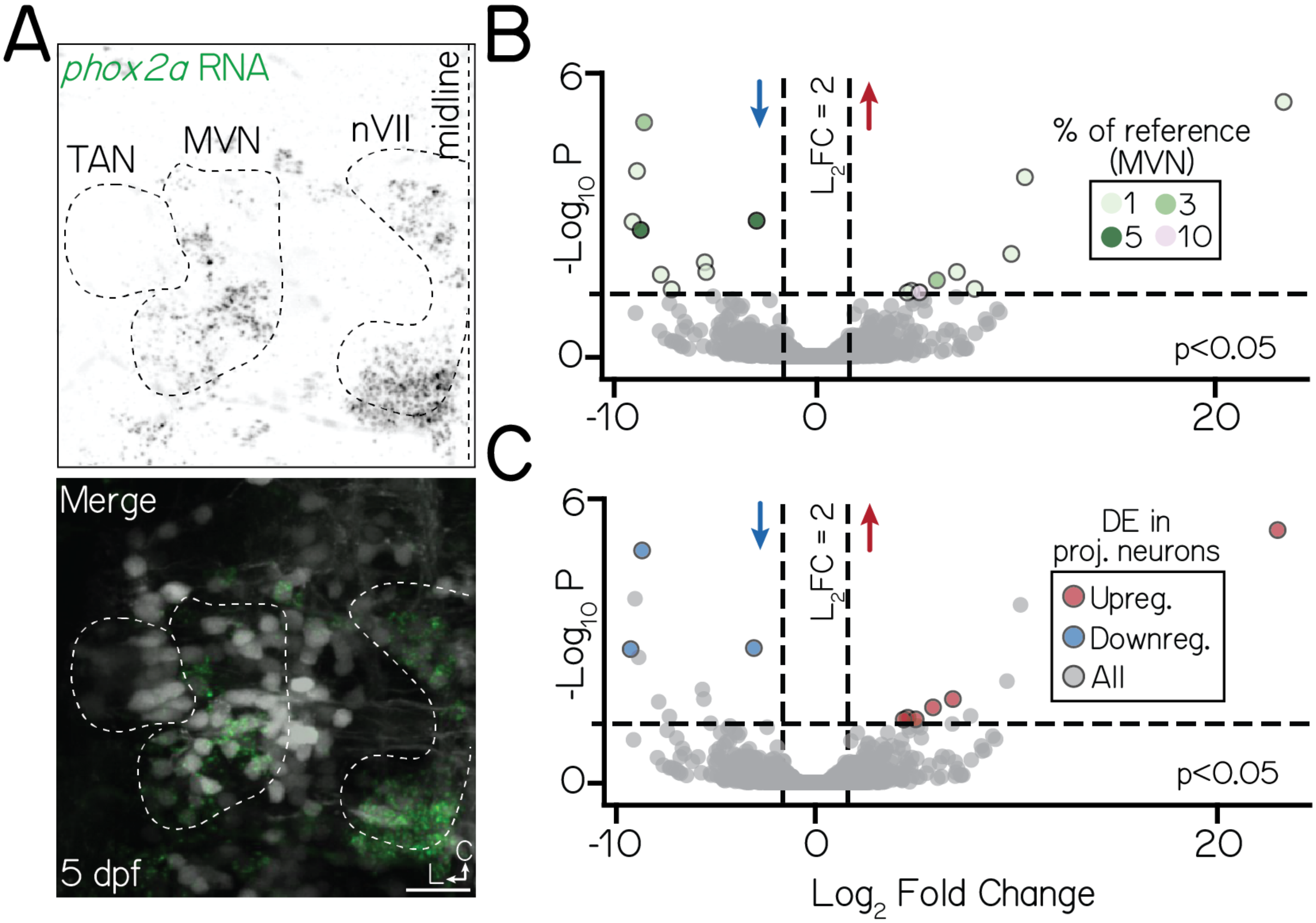

Figure S6: *phox2a* expression in the medial vestibular nucleus may underscore differential gene expression phenotypes in bulk data. Associated with Figure 5.
(A) Fluorescent *in situ* hybridization against *phox2a* in a 5 dpf larvae (axial view). Top panel shows *phox2a* RNA (green); bottom panel, merge with neurons visualized with *Tg(isl1:GFP);Tg(-6.7Tru.Hcrtr2:GAL4-VP16(;Tg(UAS-E1b:Kaede)* (grey). White dashed lines outline three nuclei of interest: projection neurons in the tangential nucleus (TAN), the medial vestibular nucleus (MVN), and the facial nucleus (nVII). All scale bars, 20 µm.
(B) Volanco plot showing differentially expressed genes in medial vestibular nucleus neurons between control and *phox2a* null larvae at 3 dpf. Dashed lines represent significance cutoffs: horizontal line, p>0.05; vertical line, Log_2 Fold Change > 2.0. Each circle is a gene. Genes to the left and right of 0 on the horizontal axis show downregulated and upregulated genes, respectively. Colors indicate percent of reference medial vestibular neurons (Methods) that express a given gene. Grey-colored genes are below both significance thresholds.
(C) Same data as Figure S6B. Color shows genes that are differentially expressed in both medial vestibular nucleus neurons and projection neurons.
